# Differences in the burden of pediatric pancreatitis across countries and regions: One size does not fit all

**DOI:** 10.1002/ueg2.12665

**Published:** 2024-11-07

**Authors:** Chinenye Rebecca Dike, Sohail Z. Husain

**Affiliations:** ^1^ Division of Pediatric Gastroenterology, Hepatology and Nutrition Department of Pediatrics The University of Alabama at Birmingham Heersink School of Medicine Birmingham Alabama USA; ^2^ Division of Pediatric Gastroenterology, Hepatology, and Nutrition, Department of Pediatrics Stanford University Palo Alto California USA

Pancreatitis is an exquisitely painful, life‐threatening inflammatory disorder of the pancreas that in children has been understudied and underappreciated.^1^ The three subtypes of pancreatitis, which in children (as opposed to adults) mostly follow a continuum, are acute pancreatitis (AP), acute recurrent pancreatitis (ARP), and chronic pancreatitis (CP).^2^ In the last decade, there has been greater awareness about the burden of pediatric pancreatitis. The reported incidence of AP in children varies across countries, ranging from 2.83/100,000 persons in Taiwan^3^ to a nearly six‐fold higher incidence of 12.3/100,000 persons in the United States (US).^4^ While the incidence in Taiwan appears to be rising,^3^ there is a leveling out of the incidence in the US.^4^ Despite laudable efforts among pediatric pancreatologists to publish consensus management guidelines for pancreatitis,^5^ studies continue to indicate variable practices, for example, in opioid versus non‐opioid analgesic use during AP admissions^6^ or in the utilization of therapeutic endoscopic retrograde cholangiopancreatography for pediatric CP.^7^

In the current issue of the *UEG Journal*, Liu et al.^8^ leveraged a longstanding dataset of global disease burden that was developed just over 30 years ago by the World Bank and the World Health Organization and is now housed in the Institute for Health Metrics and Evaluation at the University of Washington, in Seattle‐‐called the Global Burden of Disease (GBD) database.^9^ The GBD analyzes the health impact of over 100 injuries and diseases and 43 health‐related indicators, while also introducing the disability‐adjusted life year as a measure of disease burden.^9^ The most recent version of the GDB was released in 2019, and it contains information from 204 countries and geographic territories which are grouped into 21 regions and 7 super regions. The richness of this dataset makes it ideal to study measures of disease frequency and burden across regions and countries.

Liu et al.^8^ exploited the fact that the GBD includes pancreatitis, categorized according to age, and, to our knowledge, it is the first report to systematically review the global burden of pediatric pancreatitis across regions and countries. The authors reported that the global age standardized incidence rate for pediatric pancreatitis was 7.72/100,000 persons while the age standardized prevalence rate was 3.95/100,000 persons. The data suggest an overall increasing trend in incidence and prevalence rates, while there is a decline in mortality and disability.^8^ By extrapolating from a 30 year trend, the authors predict a continued rise in overall prevalence, with an overall decline in incidence, mortality, and disability rates. When parsing out the data, there were several additional findings of interest. Eastern Europe as a region had the highest incidence, and it had an increasing trend in mortality and disability. The US had the highest increase in prevalence over the 30 years, while Singapore had the highest increase in incidence. Among the pediatric age groups, the highest incidence, prevalence, mortality, and disability rates were in older children (ages 15–19 years). Among sociodemographic groups, the highest incidence was in low‐middle index regions reflecting educational status, fertility rate and income per capita.^8^

The findings of this recent study raise several more questions. Is the increasing incidence in low sociodemographic index regions due to an increased awareness of pediatric pancreatitis, resulting in greater testing? What are the trends specifically in the subtypes of AP, ARP, and CP? Incidence is a more relevant index for AP and ARP to track, since they are associated with acute events, while prevalence is more relevant to CP, since the disease is defined as a chronically painful, debilitating affliction. Unfortunately, the GBD lumps together the three subtypes, thus making trends in incidence versus prevalence more challenging to interpret. Are the baseline differences among regions for pediatric pancreatitis from the GBD, or the trends within regions, associated with genetic predisposition or environmental exposures, for example, from nutritional deficiencies,^10^ that are implicated with pancreatitis susceptibility? While the GBD has inherent limitations, Liu et al.^8^ are to be commended for utilizing the resource to decipher changing trends in pediatric pancreatitis in various countries and global regions. By overlaying additional datasets, the work may pave the way for further analysis of the factors related to these trends and their implications for policy and intervention.

## Data Availability

Data sharing not applicable to this article as no datasets were generated or analyzed in this editorial.
